# One Biosurfactant-Producing Bacteria *Achromobacter* sp. A-8 and Its Potential Use in Microbial Enhanced Oil Recovery and Bioremediation

**DOI:** 10.3389/fmicb.2020.00247

**Published:** 2020-02-19

**Authors:** Zhenshan Deng, Yingying Jiang, Kaikai Chen, Jing Li, Chaochao Zheng, Fei Gao, Xiaodong Liu

**Affiliations:** College of Life Sciences, Yan’an University, Yan’an, China

**Keywords:** microbial enhanced oil recovery, crude oil bioremediation, oil viscosity, biosurfactant, *Achromobacter*

## Abstract

Biosurfactant plays an important role in bioremediation of crude oil contamination and microbial enhanced oil recovery (MEOR). In the present study, a salt-tolerant, biosurfactant-producing bacterium, designated A-8, was isolated from wastewater contaminated with petroleum collected from the Changqing reservoir in China. A phylogenetic analysis based on the 16S rRNA sequence suggests that strain A-8 belongs to the genus *Achromobacter*. The optimal growth conditions for strain A-8 in mineral salt (MS) medium were 30°C, pH 7, and 10 g/L NaCl, while the optimal conditions for biosurfactant production in a fermentation medium were 40–45°C, pH 7, and more than 70 g/L NaCl. Better biosurfactant production was obtained from strain A-8 when edible oil and liquid paraffin were used as carbon sources and when (NH_4_)_2_SO_4_ was used as an inorganic nitrogen source compared with other tested carbon and nitrogen sources. The biodegradation of petroleum in MS medium in different optimized conditions reached 56.23–73.87% for 20 days. The biodegradation of petroleum, together with the production of organic acid and biosurfactant, decreased the viscosity of petroleum by about 45%. The decrease in petroleum viscosity and the biodegradation of petroleum suggest the potential use of strain A-8 for MEOR and bioremediation of petroleum-contaminated environments.

## Introduction

As a non-renewable resource, petroleum will gradually decrease with its continuous exploration. Three methods are usually used for petroleum exploration in oil reservoir fields: primary (5–10%), secondary (30–40%), and tertiary, which is also called enhanced oil recovery (EOR) (Datta et al., 2018; Geetha et al., 2018). After primary and secondary exploration, approximately 50% of the petroleum remains underground. Thus, EOR is necessary to improve the use of petroleum in oil reservoir fields. EOR includes physical, chemical, and biological methods (Sen, 2008; Li et al., 2011; Mozhdehei et al., 2019). Physical and chemical methods mainly aim to decrease the viscosity of the petroleum to enhance petroleum recovery. However, these methods have their own limitations, such as high cost, environmental unfriendliness, and complicated operation. Thus, biological methods, which are environmentally friendly, low in cost, and easy to operate, have always attracted significant attention (She et al., 2019). Microbial enhanced oil recovery (MEOR) is the main biological method. MEOR mainly enhances oil recovery by using the life activities of microorganisms. The degradation of petroleum and the production of biosurfactant are the two key mechanisms by which microbes can enhance oil recovery (Geetha et al., 2018). Therefore, the isolation and identification of crude-oil-degrading and biosurfactant-producing microorganisms are important parts of MEOR.

To date, numerous bacterial strains that can degrade petroleum or produce biosurfactant have been isolated from different oil-contaminated environments worldwide. Such strains include *Chelatococcus daeguensis* HB-4 from Baolige Oilfield in China (Ke et al., 2019), *Rhodococcus erythropolis* OSDS1 isolated from a solid waste management unit (SWMU) contaminated with petroleum hydrocarbons and heavy metals in the United States (Xia et al., 2019), *Bacillus subtilis* RI4914 from an oil field in Brazil (Fernandes et al., 2016), *Bacillus licheniformis* from oil-contaminated water samples in Egypt (El-Sheshtawy et al., 2015), *Pseudomonas putida* DB1 and *Bacillus cereus* DB2 isolated from petroleum-contaminated soil in India (Vinothini et al., 2015), and *B. subtilis* B30 from oil-contaminated soils in Oman (Al-Wahaibi et al., 2014). As an important part of MEOR and bioremediation, biosurfactant-producing strains have been studied under different conditions (e.g., pH, temperature, salinity, and nutrition) to find the optimal conditions for biosurfactant production. In some cases, MEOR or bioremediation must occur in relatively high-salt and high-temperature conditions (Nicholson and Fathepure, 2004). Thus, salt-tolerant and thermotolerant biosurfactant-producing bacteria are needed. Previously, Hua et al. (2010) introduced a salt-tolerant *Enterobacter cloacae* mutant that could degrade petroleum under high-salt conditions at a 7.5% NaCl concentration.

In the present study, the salt-tolerant bacterial strain *Achromobacter* sp. A-8 was isolated from wastewater contaminated with petroleum. The growth conditions and petroleum degradation properties were systematically characterized. The biosurfactant production of strain A-8 was analyzed under different conditions in terms of carbon sources, nitrogen sources, temperature, salinity, and pH. The emulsification and decreased viscosity of crude oil by biosurfactant were also characterized. The understanding of the growth and biosurfactant production conditions of strain *Achromobacter* sp. A-8 could support the application of the strain in MEOR and crude oil bioremediation.

## Materials and Methods

### Chemicals and Reagents

Crude-oil-contaminated water samples were collected from the Changqing reservoir located in Yan’an, north of Shaanxi Province, and used for bacteria isolation. Crude oil was provided by a refinery corporation from Yan’an, Shaanxi Province. The crude oil mainly consisted of n-alkanes. All chemicals used for bacterial culture and crude oil measurement were of analytical grade or better and purchased from Sinopharm Chemical Reagent Co., Ltd., China.

Luria–Bertani (LB) medium contained 10.0 g of peptone, 5.0 g of yeast extract, and 10.0 g of NaCl per liter. Mineral salt (MS) medium contained 10.0 g of KH_2_PO_4_, 2.0 g of NaNO_3_, 1.0 g of (NH_4_)_2_SO_4_, 0.3 g of MgSO_4_, 4.0 g of Na_2_HPO_4_, and 5.0 g of NaCl per liter. The enrichment medium was prepared by adding 20 g of crude oil to 1 L of MS medium. The isolation medium was prepared by adding 20 g of agar powder to the enrichment medium. The fermentation medium contained 10.0 g of glucose, 2.5 g of (NH_4_)_2_SO_4_, 10.0 g of KH_2_PO_4_, 4.0 g of Na_2_HPO_4_, and 0.3 g of MgSO_4_ per liter. All media were adjusted from the original pH to 7 using 1.0 M NaOH or 1.0 M HCl.

### Bacteria Isolation and Identification

For bacteria isolation, an enrichment culture procedure was conducted. Briefly, 5 mL of the wastewater sample was added to 100 mL of the enrichment medium and cultured at 30°C and 170 rpm for 5 days. Then, 5 mL of the enriched sample was added to 100 mL of the enrichment medium and cultured for another 5 days. The procedure was repeated three times. The last enrichment samples were used for bacteria isolation by the dilution plating method on isolation medium agar plates.

The purified isolates were cultured in LB medium to obtain a single colony. Then, colony PCR was used to obtain the 16S rRNA sequence. In brief, about 2 μL of bacteria was added to a 50 μL 2 × T5 Super PCR Mix (Colony) system (TSINGKE Biological Technology, Beijing, China) and then amplified and sequenced using the primers 27F (5′-AGAGTTTGATCCTGGCTCAG-3′) and 1492R (5′-GGTTACCTTGTTA CGACTT-3′) was previously described (Hu et al., 2015). The 16S rRNA gene sequence was submitted to GenBank and blasted using EzBioCloud (Yoon et al., 2017). The 16S rRNA gene sequence was analyzed using MEGA version 5.1 with closely related strains, and a phylogenetic tree was constructed using the neighbor-joining method (Tamura et al., 2011).

### Growth Under Different Conditions

The isolates were grown under different conditions [i.e., different temperatures (25, 28, 31, 34, 37, and 40°C), pH (5.0, 6.0, 7.0, 8.0, 9.0, and 10.0), and salt concentrations (5, 10, 20, 30, 40, and 50 g/L)] in MS medium, and growth was then measured. For tests under different temperatures, the pH of the medium was adjusted to 7, and the additional salt concentration was 0. For tests under different pH, the temperature was set to 34°C, and the additional salt concentration was 0. For tests under different salt concentrations, the temperature was set to 34°C, and the pH of the medium was adjusted to 7. All tests were cultured at 170 rpm for 1 day, and then bacterial growth was determined by measuring the optical density (OD) at 580 nm (Mahler et al., 2000).

### Crude Oil Degradation Properties Under Different Conditions

The crude oil degradation efficiency of the isolate was tested under different original pH values (3.0, 4.0, 5.0, 6.0, 7.0, 8.0, and 9.0, with a crude oil concentration of 5 g/L and salt concentration of 20.0 g/L), different original crude oil concentrations (5, 10, 15, 20, 30, 40, and 50 g/L, with pH 7.0 and a salt concentration of 20.0 g/L), and different original salt concentrations (0, 10, 15, 20, 30, 40, 50, and 60 g/L, with pH 7.0 and a crude oil concentration of 5 g/L). All tests were conducted using 100 mL of MS medium with 1 mL of the inoculate in a 250 mL flask at 170 rpm and 30°C. The crude oil degradation rate was detected after 20 days. The crude oil was extracted by CCl_4_ and dried using Na_2_SO_4_, and the concentration was measured by an infrared spectrometer oil content analyzer (OIL 480, Beijing Chinainvent Instrument Co., Ltd., China).

### Oil Displacement Test

Oil displacement tests were conducted to determine the ability of the biosurfactant to form a clear zone and to measure biosurfactant production by the isolate under different conditions. The bacteria were first cultured in the fermentation medium as the inoculum. The influence of carbon sources on biosurfactant production was tested by adding 5 mL of the inoculum to a modified fermentation medium (without glucose) containing different carbon sources (including starch, sucrose, glucose, edible oil, and liquid paraffin at a concentration of 20 g/L). The fermentation medium without nitrogen sources was used to investigate the impact of different nitrogen sources [including peptone, NaNO_3_, (NH_4_)_2_SO_4_, and NH_4_Cl at a concentration of 10 g/L, with edible oil as the sole carbon source] on biosurfactant production. The effects of temperature (26, 30, 35, 40, 45, 50, 55, and 60°C) and pH (3.0, 4.0, 5.0, 6.0, 7.0, 8.0, and 9.0) on biosurfactant production were investigated using the fermentation medium, with edible oil as a carbon source and (NH_4_)_2_SO_4_ as a nitrogen source. The effect of salt concentration (5, 10, 20, 30, 40, 50, 60, 70, 100, 200, 300, and 400 g/L) on biosurfactant production was investigated using the fermentation medium, with edible oil as a carbon source and (NH_4_)_2_SO_4_ as a nitrogen source at 45°C and pH 7.0. Bacteria fermentation was maintained for 3 days at 170 rpm. The fermentation products were collected by centrifugation at 8000 rpm, and the supernatant was used to test the oil displacement ability of the biosurfactant. Distilled water (100 mL) was poured into a Petri dish, and liquid paraffin containing 5 g/L Sudan III dye was used to form a thin layer of oil on the water surface. The supernatant (50 μL) was added to it, and the diameter of the clear zone was measured immediately. The diameter of the clear zone formed on the surface of the water represents the biosurfactant oil displacement activity (Morikawa et al., 1993).

### Surface Tension and pH of the Fermentation Solution

The bacteria were cultured in the fermentation medium at 30°C and 170 rpm for 5 days. Then, the cells were separated from the fermentation solution by centrifugation at 12,000 rpm for 10 min. The supernatant was collected and used for surface tension and pH measurements. The surface tension was measured using a fully automatic tensiometer JK99B (Shanghai Wang Xu Electric Co., Ltd., China) by the du Nouy ring method (Pandey and Pattanayek, 2013). The pH was measured using a pH meter PHS-3C (INESA Scientific Instrument Co., Ltd., China).

### Emulsification and Viscosity Decrease Test

The fermentation supernatant was prepared as described in section “Surface Tension and pH of the Fermentation Solution.” The emulsification activity (E24) was measured at room temperature as follows: the cell-free supernatant was mixed with equal volumes of crude oil and vortexed for 5 min and then settled at room temperature for 24 h (Lai et al., 2009). The E24 was calculated by the following equation:

(1) E24(%)=(totalheightoftheemulsifiedlayer)/(total⁢height⁢of⁢the⁢liquid⁢layer)× 100

The inoculum was added to the crude oil at a concentration of 2.5% (v/v) and cultured at 30°C and 170 rpm for 2 weeks. The viscosity of the original crude oil and the treated crude oil were measured using a viscometer NDJ-5S (Shjingmi Instrument Co., Ltd., China).

### Analytical Methods

All experiments were conducted three times. The data shown in the corresponding figures and tables represent the means values and the standard deviations. Statistical analysis was performed using GraphPad Prism 8 software. One-way analysis of variance (ANOVA) was used to compared the data.

## Results and Discussion

As one of the most promising green-technologies in oil recovery, MEOR can be potentially used with low operating cost and environmental friendly. The microbial metabolites, such as biosurfactant and organic acid are reported as key players in MEOR (Voordouw, 2011). In the present study, a salt-tolerant and biosurfactant-producing strain was isolated and the biosurfactant production under different conditions were tested.

### Bacteria Identification

A total of 10 strains were isolated from the crude-oil-contaminated wastewater by enrichment culture and dilution plate. Most of the bacteria strains were belonged to genus *Bacillus*, especially species *B. cereus*, which already had been widely reported involved in crude oil degradation (Banerjee and Ghoshal, 2010; Janaki et al., 2016). An oil displacement test was used to preliminary determination of the biosurfactant production of each strains and strain A-8 possessed the largest clear zone. Thus strain A-8 was selected for biosurfactant production and crude oil degradation tests while the other strains were stored in a 20% glycerol storage solution at −80°C.

**FIGURE 1:**
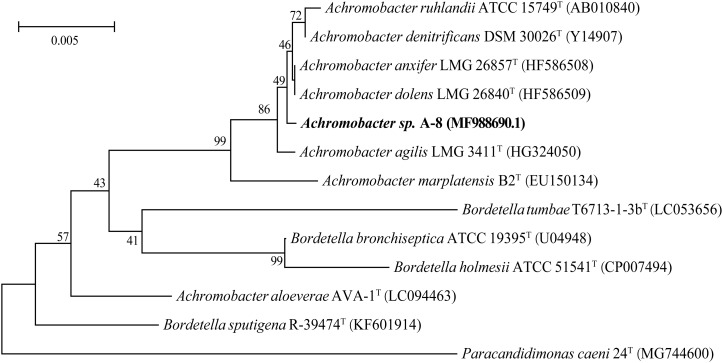
Neighbor-joining phylogenetic tree based on the 16S rRNA sequences of strain A-8 and closely related strains. Bar = 0.005 nucleotide substitutions per position.

Cells of strain A-8 were Gram-stain-negative, rod-shaped (about 0.5–0.6 μm wide and 1.1–1.8 μm long) (Supplementary Figure 1). Colonies on LB medium appeared to be light yellow, round, and convex with regular edges (Supplementary Figure 2). The results of the 16S rDNA sequence analysis suggest that strain A-8 possesses 99.32% sequence similarity with the strain *Achromobacter ruhlandii* ATCC 15749. The 16S rDNA sequence of strain A-8 was MF988690.1. A phylogenetic tree based on the 16S rDNA sequence of strain A-8 and the closest related strains was constructed by neighbor-joining (Figure 1). On the basis of the phylogenetic analysis results, strain A-8 is tentatively suggested to be a member of genus *Achromobacter*.

### Growth Profile of Strain A-8

**FIGURE 2:**
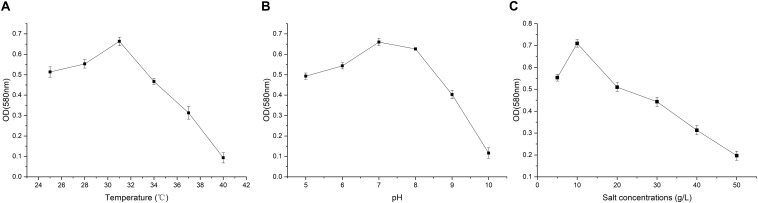
Growth profile of strain A-8 in MS medium under different temperatures **(A)**, pH **(B)** and salt concentrations **(C)**.

To be a powerful genus involved in bioremediation of crude-oil-contaminated environment, many *Achromobacter* strains were isolated and identified (Ghevariya et al., 2011; Deng et al., 2016; Hou et al., 2018; Haloi and Medhi, 2019). Understanding the optimum growth conditions of the bacteria is beneficial to the application of the strains. The growth profile of strain A-8 under different conditions was tested, and the results are shown in Figure 2. Temperature is a critical environmental factor affecting microbial growth and reproduction (Ratkowsky et al., 1982). The optimal temperature for strain A-8 was 31°C and the OD580 at 31°C was significantly higher than the other tested temperatures (*P <* 0.01). As the temperature increased or decreased from this value, the growth rate of strain A-8 decreased. The optimal pH for strain A-8 was about 7, and a slight decrease in growth was found at pH 8. The growth rate dramatically decreased when the pH exceeded 9. The OD580 of strain A-8 at pH 7 was significantly higher than the other tested pH (*P <* 0.05) except pH 8. The optimal salt concentration for strain A-8 was about 10 g/L. There was a pronounced decrease in growth in salt concentrations lower or higher than 10 g/L. After 1 day of culture, the OD580 of strain A-8 at salt concentration of 10 g/L was significantly higher than other tested salt concentrations (*P <* 0.01). Thus, the optimal growth conditions for strain A-8 in MS medium were determined to be 30°C, pH 7, and a NaCl concentration of 10 g/L. However, the results also showed that there was still marked growth for strain A-8 at a salt concentration of 40 g/L.

### Crude Oil Degradation Characteristics of Strain A-8

**FIGURE 3:**
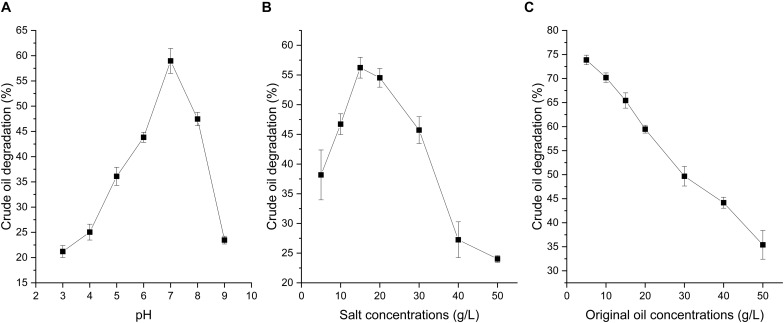
Crude oil degradation rate of strain A-8 in MS medium under different pH **(A)**, salt concentrations **(B)** and original oil concentrations **(C)**.

Soil pH, salinity, and crude oil concentration have been reported to play important roles in crude oil degradation (Mille et al., 1991; Das and Chandran, 2011). As shown in Figure 3, the crude oil degradation of strain A-8 under different pH, salinity, and original crude oil concentrations had large differences. The optimal crude oil degradation conditions were a neutral pH and a salt concentration of 15 g/L. The crude oil degradation at a neutral pH was significantly higher than the other tested pHs while at different salinity, the crude oil degradation at 15 g/L was significantly higher than the other tested salt conditions except 20 g/L (*P* < 0.05). The crude oil degradation ability decreased as the original crude oil concentration increased. The crude oil degradation dramatically decreased when the pH was higher than 8 and lower than 5, and the crude oil depletion rate decreased from its highest value of 60% to lower than 25% (Figure 3A). For the influence of salt concentration on crude oil degradation, when the salt concentration was higher than 40 g/L, the crude oil depletion rate decreased to lower than 30%. However, at the salt concentration of 30 g/L, the crude oil depletion rate still reached about 45%. Thus, strain A-8 was suitable for the biodegradation of crude oil contamination in high-salinity environments.

High concentrations of the original crude oil inhibited the growth of microorganisms. The oil degradation rate at the original crude oil concentration of 5 g/L was significantly higher than the other tested original crude oil concentrations (*P <* 0.05). As the original crude oil concentration increased from 5 to 50 g/L, the degradation rate of crude oil dramatically decreased from 73.9 ± 1.0% to 35.4 ± 3.0% (Figure 3C). Some other microorganisms that can degrade crude oil have shown similar characteristics (Sathishkumar et al., 2008).

### Potential Ability of Strain A-8 in MEOR

Microbial enhanced oil recovery was first proposed by Beckman (1926), but this technology was slowly developed during a long period of time due to the limits of science and technology (Brown, 2010). As a key point of MEOR, the finding of proper microorganisms had attracted a lot of concentrations and many bacteria strains were isolated and characterized in laboratory (Al-Wahaibi et al., 2014; Fernandes et al., 2016; Zhang et al., 2016; Ke et al., 2019). The field tests were important for MEOR from laboratory experiments to industry application. *Brevibacillus brevis* and *B. cereus* were selected and conducted field tests in Daqing Oilfield and the average crude oil production was increased (Guo et al., 2007). Clostridium strains were also conducted filed tests in central Oklahoma and the oil production increased by about 250% (Grula et al., 1985). However, the physicochemical properties of the wells played essential roles in the MEOR (She et al., 2019). Therefore, it is important to understand the growth conditions of the isolates and the physicochemical characteristics of the oil wells.

**FIGURE 4:**
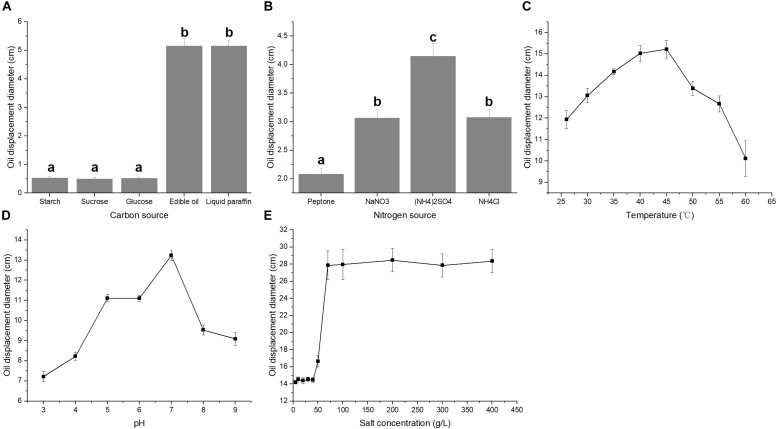
Biosurfactant production of strain A-8 in a fermentation medium with different carbon sources **(A)**, nitrogen sources **(B)**, temperatures **(C)**, pH **(D)** and salt concentrations **(E)**. The significance of differences is shown at the *P* < 0.05 level (ANOVA). Different lowercase letters mean significant differences between two columns.

The fermentative production of biosurfactant, acid matter and gas during the metabolism of microorganisms could decrease the viscosity and increase the fluidity of crude oil (She et al., 2019). In the present study, the biosurfactant and acid matter production of strain A-8 were tested. The formation of clear zone on oil surface is an important feature of biosurfactants, which provides an indicator to evaluate the potential ability of biosurfactant-producing bacteria (Youssef et al., 2004). Both nutrient types and culture conditions affected the oil displacement index. Similar to the strain *Pseudomonas aeruginosa* J4 (Wei et al., 2005), strain A-8 formed larger oil displacement clear zones, indicating better biosurfactant production, when edible oil and liquid paraffin were used as the carbon sources compared with the other three carbon sources (Figure 4A). However, glucose was found to be the best carbon source for biosurfactant production in some other strains (Batista et al., 2006). Strain A-8 formed larger oil displacement diameters when (NH_4_)_2_SO_4_ was used as the nitrogen source compared with the other three nitrogen sources (Figure 4B). A similar phenomenon was observed in the strain *B. subtilis* RI4914, for which NH_4_NO_3_ was the best nitrogen source for biosurfactant production (Fernandes et al., 2016). The largest oil displacement diameter in the temperature tests occurred at 40–45°C, while the best pH for biosurfactant production was about 7 (Figures 4C,D). The osmotic pressure of culture medium increases greatly with the increase of salt concentration and it will greatly affect the growth and metabolism of bacteria (Greenway and Osmond, 1972). The oil displacement diameter for strain A-8 at salt concentrations between 5 and 50 g/L was about 15 cm. However, the oil displacement diameter sharply increased when the salt concentration reached 70 g/L, and it remained about 27 cm with salt concentrations between 70 and 400 g/L (Figure 4E). The results reveal that the high-salinity condition is suitable for the production of biosurfactant by strain A-8. The salt-tolerant *E. cloacae* mutant showed higher exopolysaccharide (EPS) production in 9% NaCl relative to the control, which implied the high biosurfactant production of this strain under high-salt conditions (Hua et al., 2010).

The fermentative production of biosurfactant, acid matter, and gas during the metabolism of microorganisms can decrease the viscosity and increase the fluidity of crude oil (She et al., 2019). The metabolic production of strain A-8 was able to reduce the pH and surface tension of the fermentation solution (Table 1), which demonstrates the potential use of A-8 for MEOR. The reduced surface tension also suggests the production of biosurfactant by A-8. The crude oil viscosity was reduced from 2136 ± 41 to 1176 ± 79 mPa⋅s (mean ± SD) after treatment with A-8 (Table 1). The dramatically decreased viscosity also indicates the potential use of A-8 for MEOR. The emulsification of the fermentation solution of strain A-8 was 65.4 ± 2.1% (mean ± SD). The emulsification caused by biosurfactant makes a great contribution to the bioremediation of crude oil contamination and MEOR. The E24 of strain *Achromobacter* sp. HZ01 in cocoanut oil could reached to about 90% (Deng et al., 2016). The complex contents of crude oil might explained the lower E24 of strain A-8 than HZ01.

**TABLE 1:** Summary of changes in crude oil viscosity, fermentation solution pH, and fermentation solution surface tension.

Crude oil viscosity (mPa⋅s)		Fermentation solution pH		Fermentation solution surface tension (mN/m)	
Original	Final	Original	Final	Original	Final
2136 ± 41	1176 ± 79	7 ± 0.1	5.8 ± 0.2	73.7 ± 0.2	62.8 ± 1.4

## Conclusion

A salt-tolerant crude-oil-degrading bacterial strain (A-8) was isolated from wastewater contaminated with crude oil. The strain belonged to the genus *Achromobacter* based on 16S rRNA sequence analysis results. The high oil degradation rate in MS medium under suitable conditions implied the potential use of strain A-8 for the bioremediation of crude-oil-contaminated environments. Our results showed that biosurfactant produced by strain A-8 can efficiently reduce surface tension, and has high oil-displacement efficiency. In addition, the biosurfactant production of strain A-8 under high-salt conditions was much higher than that under low-salt conditions. These results implied the potential use of strain A-8 for the MEOR.

## Data Availability Statement

All datasets generated for this study are included in the article/Supplementary Material.

## Author Contributions

ZD and XL designed the study. KC, FG, and JL conducted the research. YJ and CZ analyzed the data. XL and YJ wrote the manuscript.

## Conflict of Interest

The authors declare that the research was conducted in the absence of any commercial or financial relationships that could be construed as a potential conflict of interest.
