# Supplementary material for: One Biosurfactant-Producing Bacteria Achromobacter sp. A-8 and Its Potential Use in Microbial Enhanced Oil Recovery and Bioremediation
Source: Front Microbiol. 2020 Feb 19;11:247. doi: 10.3389/fmicb.2020.00247 (PMC7042313; doi:10.3389/fmicb.2020.00247)
Supplement: Supplementary file 1 [file Data_Sheet_1.PDF]

Supplementary Material

1 Supplementary Figures and Tables

1.1 Supplementary Figures

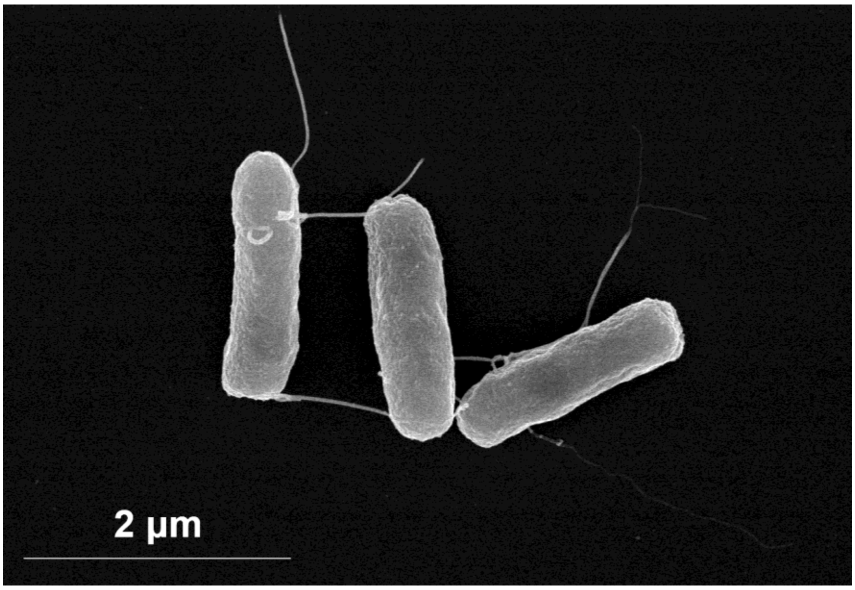

Supplementary Figure 1. Scanning electron micrograph of strain A-8. Bar, 2 µm.

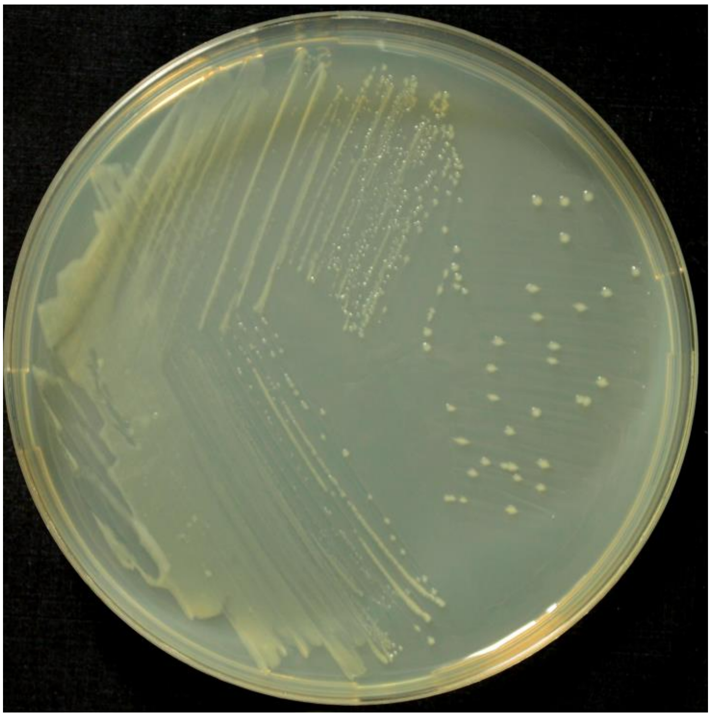

Supplementary Figure 2. Morphology of strain A-8 grown on LB medium for 2days at 30℃.
